# The Variation Patterns of the Martensitic Hierarchical Microstructure and Mechanical Properties of 35Si2MnCr2Ni3MoV Steel at Different Austenitizing Temperatures

**DOI:** 10.3390/ma17051099

**Published:** 2024-02-28

**Authors:** Zhipeng Wu, Chao Yang, Guangyao Chen, Yang Li, Xin Cao, Pengmin Cao, Han Dong, Chundong Hu

**Affiliations:** 1 School of Materials Science and Engineering, Shanghai University, Shanghai 200444, China; reay777@126.com (Z.W.); alphayangchao@shu.edu.cn (C.Y.); cgybless1@shu.edu.cn (G.C.); gody1106040057@163.com (Y.L.); donghan@shu.edu.cn (H.D.); 2 Zhejiang Institute of Advanced Materials, Shanghai University, Jiaxing 314100, China; caoxin961113@163.com; 3 Zhongyuan Special Steel Co., Ltd., Jiyuan 459000, China

**Keywords:** ultra–high strength steel, mechanical properties, microstructure

## Abstract

This study investigates the influence of varying austenitizing temperatures on the microstructure and mechanical properties of 35Si2MnCr2Ni3MoV steel, utilizing Charpy impact testing and microscopic analysis techniques such as scanning electron microscopy (SEM) and electron backscatter diffraction (EBSD). The findings reveal that optimal combination of strength and toughness is achieved at an austenitizing temperature of 980 °C, resulting in an impact toughness of 67.2 J and a tensile strength of 2032 MPa. The prior austenite grain size initially decreases slightly with increasing temperature, then enlarges significantly beyond 1100 °C. The martensite blocks’ and packets’ structures exhibit a similar trend. The proportion of high–angle grain boundaries, determined by the density of the packets, peaks at 980 °C, providing maximal resistance to crack propagation. The amount of retained austenite increases noticeably after 980 °C; beyond 1200 °C, the coarsening of packets and a decrease in density reduce the likelihood of trapping retained austenite. Across different austenitizing temperatures, the steel demonstrates superior crack initiation resistance compared to crack propagation resistance, with the fracture mode transitioning from ductile dimple fracture to quasi–cleavage fracture as the austenitizing temperature increases.

## 1. Introduction

Ultra–high–strength steels are crucial in aerospace applications and are extensively used in the manufacturing of armor–piercing shell bodies. With advancing technological demands for penetration depth and initial shell velocity, these shell bodies require heightened strength to withstand the load. However, as the strength of steel reaches a certain threshold, its toughness is significantly compromised. This contradictory tendency escalates with increased strength, adversely affecting service safety and limiting the application of ultra–high–strength steels in armor–piercing shells. To achieve a balanced match between strength and toughness in ultra–high–strength steels, traditional high–alloy versions largely depend on the inclusion of precious metal elements like cobalt and nickel to enhance toughness, resulting in high production costs. Therefore, from an economic perspective, they are no longer suitable for large–scale industrial production. On the contrary, low–alloy ultra–high–strength steels represent an important development direction due to their relatively low cost. The AISI 4130, AISI 4140, and AISI 4340 alloy steel series in the United States are typical representatives of early low–alloy ultra–high–strength and –toughness steels [1,2]. Among them, AISI 4130 was the earliest developed ultra–high–strength alloy steel. To achieve the best combination of strength and toughness, this steel is often subjected to tempering treatment. However, its yield strength and tensile strength are 880 and 980 MPa, respectively, which do not meet the requirements of ultra–high–strength alloy steels. Therefore, on the basis of 4130 steel, the carbon content is increased to 0.4%, and small amounts of Ni and Mo elements are added to form 4340 steel. Most of the subsequently developed low–alloy ultra–high–strength steels have been continuously improved based on the AISI 4130 and AISI 4340 alloy steel series. Due to the higher carbon content in low–alloy carbon steels, their strength is high after quenching, but their ductility is poor. Therefore, Krauss [3] conducted low–temperature tempering treatment (150–200 °C) on martensitic carbon steel and low–alloy carbon steel. Their results showed that the strength of low–temperature tempered martensite is related to the dynamic work hardening of dislocations and transition carbides in martensite crystals, which is determined by the carbon content. In steel with 0.5% C, when second–phase particles are dispersed in the tempered martensite matrix, toughness notches form on them and lead to plastic fracture. In low–temperature tempered martensitic steels with more than 0.5% C, brittle intergranular fractures easily occur along the original austenite grain boundaries. Therefore, the carbon content in low–alloy ultra–high–strength steels generally should not exceed 0.5%, and appropriate low–temperature tempering treatment is usually required to improve their toughness. In order to further enhance the toughness of low–alloy ultra–high–strength steels, Chang and Smith [4] studied the effect of Si element on the tempering hardness and microstructure of martensite and discovered the beneficial role of Si in low–alloy ultra–high–strength steels, laying the foundation for the development of 300 M steel. In 1952, the International Nickel Company in the United States developed and designed 300 M steel, which was developed based on AISI 4340 steel by adding 1.52.0% Si element and slightly adjusting the V content. With proper element control and heat treatment processes, the development of low–alloy ultra–high–strength steels has become increasingly mature and is now rapidly progressing towards higher strength, toughness, and lower cost. For example, Fe–0.25C–1.6Si–1.5Mn–0.5Cr–0.3Mo steel developed by the Russian researcher Tkachev [5] achieved a tensile strength of 1840 MPa and an impact energy of 87 J after quenching at 950 °C and tempering at 280 °C. These properties are comparable to those of high–alloy ultra–high–strength steels such as AerMet 100 (AerMet is a registered trade mark of CRS Holdings, Inc., Philadelphia, PA, USA), but at a significantly reduced cost.

Building on previous research, our team has developed a novel low–alloy ultra–high strength 35Si2MnCr2Ni3MoV steel, costing only one–eighth of the price of AerMet 100 steel. However, achieving optimal properties necessitates precise thermal processing. Both excessively high and low austenitizing temperatures can directly affect the distribution of alloy elements and subsequent phase transformations during quenching, particularly martensitic transformations, thus significantly influencing the material’s properties. Recent studies on common low–alloy ultra–high strength steels indicate that austenitizing temperature affects the prior austenite grain size, indirectly impacting final toughness [6]. The solid solution and martensitic transformation strengthening effects in ultra–high strength steels are also related to austenitizing temperature [7]. Austenitizing can enhance microstructural uniformity and reduce segregation [8,9,10]. Therefore, the temperature plays a significant role in affecting the outcomes of austenitizing treatment, including microstructural uniformity, solid solution strengthening, and martensitic transformation strengthening.

Based on this, the influence of different austenitizing temperatures on the microstructure and mechanical properties of 35Si2MnCr2Ni3MoV steel is analyzed in this paper. The variation patterns of the size of the martensitic multilevel structure units after different austenitizing temperatures and the transition of fracture mechanisms at different austenitizing temperatures are investigated. The aim of this analysis is to provide a theoretical basis for the rational design of heat treatment processes for 35Si2MnCr2Ni3MoV steel in industrial production.

## 2. Materials and Methods

The chemical composition of the 35Si2MnCr2Ni3MoV steel used in the experiments is presented in Table 1.

The steel was produced using a vacuum induction plus vacuum self–consumption remelting process. The ingot underwent forging heating, triple drawing, billet precision forging, and post–forging annealing, then was processed into a rod with the dimensions of φ170 mm × 80 mm.

The phase transformation points of 35Si2MnCr2Ni3MoV steel were determined using a dilatometer (DIL805A, TA, Milford, MA, USA), which guided the establishment of appropriate austenitizing and tempering temperatures.

**Figure 1:**
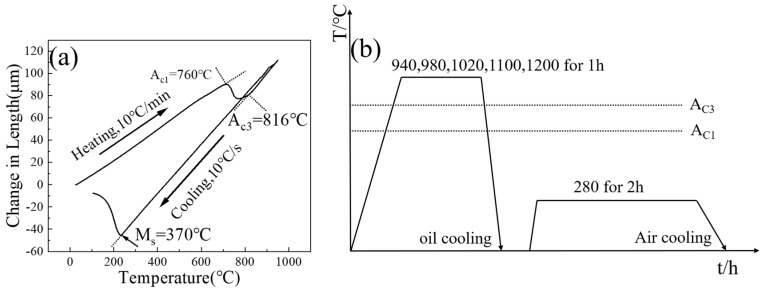
(**a**) Thermal expansion curve; (**b**) heat treatment process system.

The dilatometry samples, in a post–forging annealed state, were sized at φ4 × 10 mm. The heating rate for the measurement was set at 0.1 °C/s, and the cooling rate was set at 20 °C/s. Based on the determined transformation points of Ac1 = 760 °C, Ac3 = 816 °C, and Ms = 270 °C, the heat treatment process is depicted in Figure 1b.

The impact specimens, measuring 10 × 10 × 55 mm and featuring a 2 mm deep U–shaped notch, were utilized for room temperature impact testing using a testing machine (Instron 750MPX, Boston, MA, USA) with three samples tested at each temperature (test with reference to standard ASTM E23-2018 [11]). Metallographic samples, measuring 10 × 10 × 5 mm, were prepared and subjected to etching using a 4% nitric acid alcohol solution.

The macroscopic and microscopic morphology of impact fracture surfaces was examined using a stereomicroscope (ZEISS Stemi 508, Carl Zeiss, Jena, Germany) and a field emission scanning electron microscope (FEI Apreo 2S HiVac, Thermo Fisher Scientific Waltham, MA, USA). The martensitic substructure in the microstructure was observed and analyzed via EBSD (Bruker QUANTAX EBSD 400i e–Flash^FS^, Bruker, Billerica, MA, USA), in combination with software such as AZtecCrystal v2.1, MATLAB(R2022a), and MTEX toolbox v5.9.1.

## 3. Results and Discussion

**Figure 2:**
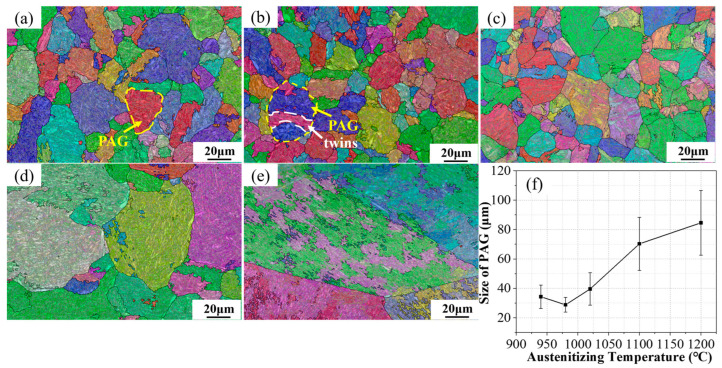
IPF map of reconstructed parent austenite grains with the parent grain boundaries in yellow and parent austenite twin boundaries in white after austenitizing at 940 °C (**a**), 980 °C (**b**), 1020 °C (**c**), 1100 °C (**d**), and 1200 °C (**e**) and the average grain size statistics (**f**).

Figure 2 presents the morphology of the prior austenite grain sizes at different austenitizing temperatures, reconstructed using AZtecCrystal software. By means of the intersecting line method (refer to standard ASTM E112–13 [12]), the average grain size after reconstruction was estimated. (We understand that Figure 2 represents a cross–section of mostly uniform grains. Some display the whole diameter, while others are just a small part of the sphere cross–section, thus appearing as small grains. This was taken into account in the estimation of the average grain size). As observed from Figure 2a–e, at austenitizing temperatures of 940, 980 and 1020 °C, the average grain size does not change significantly. Twins [5] are observable within the grains at 980 °C and 1020 °C. As the temperature further increases to 1100 °C, there is a noticeable increase in grain size, and at 1200 °C, the grains undergo severe coarsening, with only the prior austenite grain boundaries being observable. With the austenitizing temperature rising from 940 °C to 1200 °C, the grain size in the steel first decreases from 31.3 μm to 27.6 μm and then increases to 85.6 μm, as specifically fitted and shown in Figure 2f. The significant increase in grain size is attributed to the enhanced movement and diffusion ability of boundary atoms with increasing temperature, accelerating the rate of grain boundary engulfment. According to the Hall–Petch formula, strength is inversely related to the prior austenite grain size [13]. Given a constant total plastic deformation, the growth of grains results in dislocation motion occurring in fewer grains, leading to uneven plastic deformation and a propensity for stress concentration. This uneven distribution of deformation can reduce plastic toughness.

**Figure 3:**
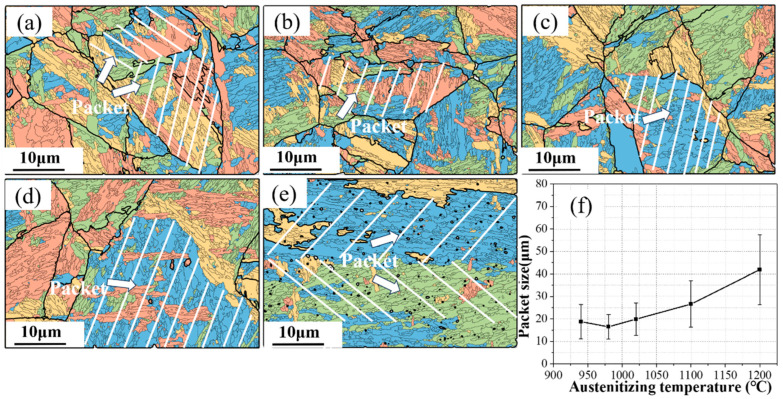
EBSD map showing the four different type of martensite packets and grain boundaries of the reconstructed austenite grains at 940 °C (**a**), 980 °C (**b**), 1020 °C (**c**), 1100 °C (**d**), and 1200 °C (**e**) and the average packet statistics (**f**).

In addition to the changes in the prior austenite grain size affecting both the strength and toughness of the alloy, research by Luo [14] suggests that the refinement of the martensitic microstructure may also play a similar role. Therefore, it is necessary to further explore the width variation of martensite blocks and packets at different austenitizing temperatures. The morphology of martensite packets was reconstructed using MATLAB and the MTEX toolbox [15]. The authors point out that typically, a complete martensitic package contains variants of four colors, which allows us to distinguish some martensitic slat packages quite well. The results are depicted in Figure 3. From Figure 3a–c, it can be observed that the size of the packet changes minimally in the 940 to 1020 °C range. However, as the austenitizing temperature increases to 1100 °C, the size of the packet notably enlarges, and further increases when the temperature reaches 1200 °C. The sizes of martensite packets at five different temperatures were statistically analyzed with 30 measurements each, and the fitted sizes are shown in Figure 3f. At austenitizing temperatures of 940, 980, 1020, 1100, and 1200 °C, the average size of the martensite packets is 18.8, 16.5, 19.9, 26.6, and 41.9 μm, respectively. As the austenitizing temperature increases from 940 °C to 1200 °C, the size of the packets first decreases and then increases, following the same trend as the prior austenite grain size.

To accurately quantify the size of martensite packets, this study referenced the relationship between the various hierarchical structures of martensite as proposed by E.I. Galindo–Nava [16].

(1) $$D_{Packet}=\sqrt{\frac{3\sqrt{3}}{8N_{p}}}D_{PAG}$$

(2) $$d_{block}=\frac{1}{N_{b}}D_{Packet}$$

This formula considers *N_b_* as the number of packets in each block, with the number of packets in each block depending on the block’s size. Typically, this ranges from two packets in blocks of about 2 μm in size to six packets in blocks exceeding 10 μm. Research by Tkachev [5] and others indicates that the average *N_b_* value in the experiments is five.

**Figure 4:**
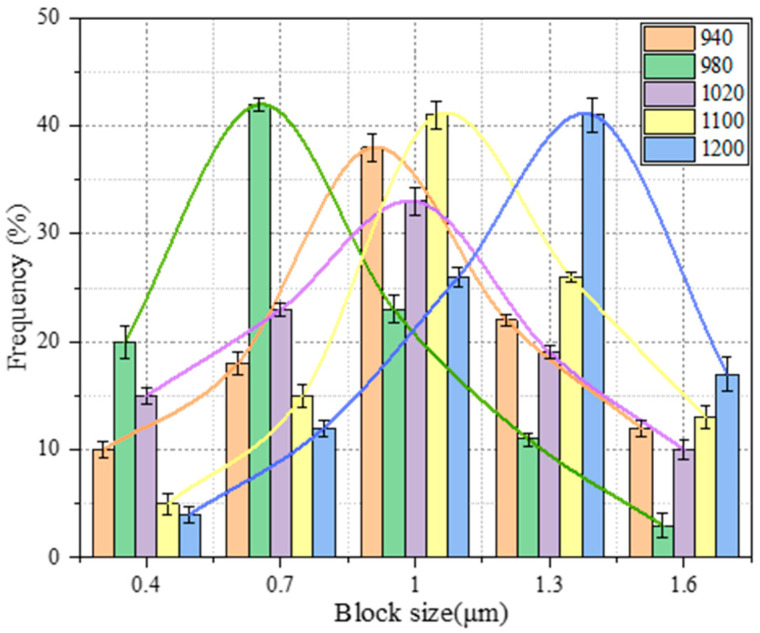
The distributions of martensite block sizes at five austenitizing temperatures. The connected lines are shown to exhibit a Gaussian distribution.

Based on this formula, the sizes of 300 packets at five different austenitizing temperatures were statistically analyzed, with the results shown in Figure 4. At these varying austenitizing temperatures, the average widths of the martensite blocks were 0.954, 0.795, 0.958, 1.081, and 1.375 μm, respectively. Although these changes in packet size were smaller compared to those in blocks, the results still confirm that the sizes of martensite blocks and packets are influenced by the size of the prior austenite grains.

**Figure 5:**
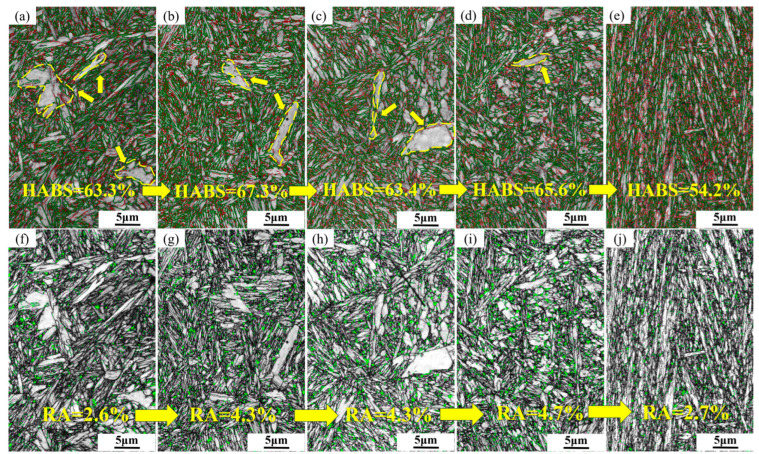
The amount of high–angle grain boundaries and retained austenite at five austenitizing temperatures. The yellow arrows and numbers represent the specific values after the change with temperature. (**a**,**f**) 940 °C; (**b**,**g**) 980 °C; (**c**,**h**) 1020 °C; (**d**,**i**) 1100 °C; (**e**,**j**) 1200 °C.

High–angle grain boundaries (HAGBs) in martensite greatly inhibit crack propagation [17]—as the crystal orientation angle increases, cracks consume more shear energy when crossing these boundaries, leading to significant deviation in the crack propagation direction at HAGBs [18,19,20]. Therefore, the proportion of high–angle grain boundaries to some extent reflects the toughness differences of steel at various austenitizing temperatures. As shown in Figure 5a–e, the proportions of HAGBs at different austenitizing temperatures were quantified, with values of 63.3%, 67.3%, 59.4%, 58.8%, and 48.2%, respectively. In these figures, red and green lines represent low–angle and high–angle grain boundaries, respectively. With increasing austenitizing temperature, the proportion of HAGBs first increases and then decreases, reaching its peak at 980 °C. This indicates that at an austenitizing temperature of 980 °C, the block size is the smallest and the interface is the most numerous, which corresponds with the previously observed trends in block size variation.

Retained austenite also impedes crack propagation in steel, and its amount can be influenced by changing the austenitizing temperature of the steel. Figure 5i, j show the amounts of retained austenite at different austenitizing temperatures. For austenitizing temperatures of 940, 980, 1020, 1100, and 1200 °C, the retained austenite contents are 2.6%, 4.3%, 4.3%, 4.7%, and 2.7%, respectively. Observations reveal that in Figure 5f–h, where massive and striated martensitic substructures are present (indicated by arrows), the distribution of retained austenite is low. As the austenitizing temperature increases, these massive and striated substructures diminish or disappear. Retained austenite is primarily distributed along high–angle and low–angle grain boundaries. With increasing austenitizing temperature, the amount of retained austenite in the steel significantly rises, related to the solubility of stable austenite–forming elements in the steel. However, at 1200 °C, the amount of retained austenite notably decreases due to severe grain coarsening at this temperature, which consequently reduces the density of the block substructure and lowers the probability of the presence of retained austenite at the block boundaries.

**Figure 6:**
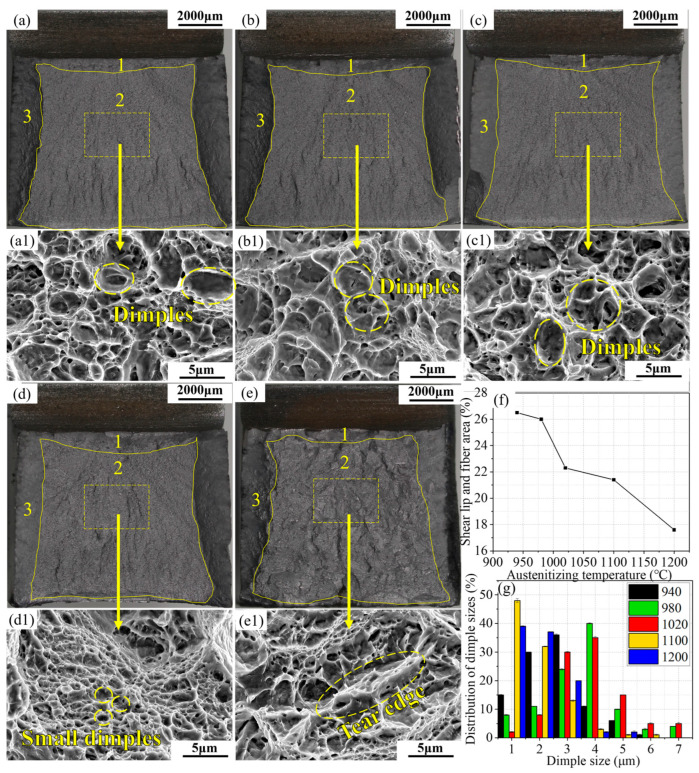
Macroscopic fracture surfaces of the Charpy U–notch specimens of the 940 °C (**a**), 980 °C (**b**), 1020 °C (**c**), 1100 °C (**d**), and 940 °C (**e**) steel. (**a1**–**e1**) correspond to the SEM images of stable crack propagation zones. The proportion of area of shear lip fiber area in five specimens is shown in (**f**) and the distributions of dimple sizes are shown in (**g**). The numbers 1, 2 and 3 correspond to the crack initiation, fast crack propagation zones and shear lip.

A rough assessment of material toughness can be performed by evaluating the proportions of the crack initiation zones (1), fast crack propagation zones (2), and shear lip (3) in the macroscopic fracture surface of steel. It is generally believed that larger proportions of the crack initiation zones and shear lip indicate better ductility and toughness of the material [21]. Figure 6 shows the impact fracture morphologies of the alloy steel at different austenitizing temperatures. The proportions of the shear lip and crack initiation zones in the impact fractures after different austenitizing temperatures were calculated using Image–Pro Plus 6.0 software, resulting in 26.5%, 26%, 22.3%, 21.4%, and 17.6%, respectively. It was found that the ductility and toughness of the material are relatively better at austenitizing temperatures of 940 °C and 980 °C.

Figure 6a1–e1 show the microscopic morphology in the rapid propagation region. Observations from Figure 6a1–c1 reveal that at austenitizing temperatures of 940 to 1020 °C, the fracture is mainly composed of dense dimples with relatively large sizes. As the temperature increases to 1100 °C (Figure 6d1), the size of the dimples significantly reduces, and at 1200 °C, clear tear ridges appear in the fracture, indicating a transition in the fracture mode of the test steel from ductile dimple fracture to quasi–cleavage fracture. The sizes of approximately 1000 dimples were measured using the linear intercept method, with the results shown in Figure 6f. At austenitizing temperatures of 940, 980, 1020, 1100, and 1200 °C, the average dimple sizes are 3.25, 3.64, 3.46, 2.24, and 2.37 μm, respectively. With increasing austenitizing temperature, the average diameter of the dimples first increases and then decreases, reaching its maximum at 980 °C. According to Hilders’ research [22], impact toughness is inversely related to the square root of the average dimple size, indicating that at an austenitizing temperature of 980 °C, the toughness is relatively improved.

**Figure 7:**
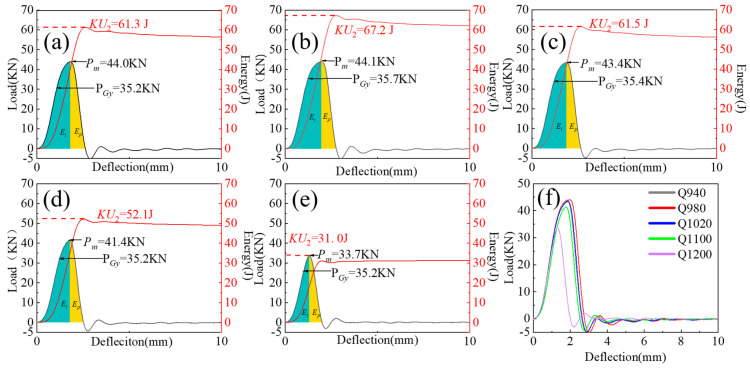
The effect of austenitizing temperature on the load–deflection curve and energy–deflection curve: (**a**) 940 °C; (**b**) 980 °C; (**c**) 1020 °C; (**d**) 1100 °C; (**e**) 1200 °C; (**f**) general overview. *E_i_* and *E_p_* represent energy to initiate crack and energy to propagate crack.

Figure 7 presents the load–deflection and energy–deflection curves obtained from oscillatory shock testing at five different austenitizing temperatures. The results show that as the austenitizing temperature increases, the impact energy absorption first rises and then falls, reaching a maximum of 67.2 J at 980 °C and decreasing by about 50% at 1200 °C. At different quenching temperatures, the proportion of *E_i_* (energy to initiate crack) is higher than *E_p_* (energy to propagate crack), indicating that the specimens have better crack initiation resistance, with *E_i_* dominating the energy absorption during the entire impact process. In many studies [23,24,25], it was noted that the ratio of crack propagation energy to initiation energy reflects the ductile–brittle fracture state of the material. At different austenitizing temperatures, the *E_p_/E_i_* ratio is <1, suggesting that the material tends to brittle fracture. When the quenching temperature reaches 1200 °C, *E_i_* and *E_p_* are 17.9 J and 13.1 J, respectively, with *E_i_* decreasing by 62% from its highest value and *E_p_* decreasing by 38%. This indicates that at 1200 °C, the crack in the impact specimen is in the most susceptible state for initiation and propagation, and its resistance to crack initiation and propagation significantly diminishes. The proportions of *E_i_* and *E_p_* are 57.7% and 42.3%, respectively, with the proportion of *E_i_* decreasing by about 20% and that of *E_p_* increasing by 20%, indicating a decrease in the dominant role of *E_i_* in the entire impact fracture process. The maximum load Pm shows a decreasing trend with increasing austenitizing temperature, dropping from 44 KN at 940 °C to 33.7 KN at 1200 °C. Dynamic yield strength reflects the level of impact toughness; the higher the dynamic yield strength, the greater the impact toughness.

The dynamic yield strength *σ_GYd_* can be calculated using the following formula [26]:

(3) $$\sigma_{GYd}=\frac{3.732P_{GY}W}{C_{GY}{\left(W-a\right)}^{2}B}$$

where *W* represents the width of the specimen (10 mm); *B* is the thickness of the specimen (10 mm); a is the notch depth (2 mm); and *C_GY_* depends on the shape of the indenter and the radius at the root of the notch, and is taken as 1.336 in this study [26]. The dynamic ultimate tensile strength σ*_UYd_* can be obtained using a similar relationship, with the formula being as follows [26]:

(4) $$\sigma_{UTSd}=\frac{\eta_{Pm}P_{m}W}{{\left(W-a\right)}^{2}B}$$

where *η_pm_* is an empirical factor depending on the ratio between shear stress and tensile stress, as well as the constraint factor at maximum load. In this study, *η_pm_* is taken as 2.929, a value referenced from calculations for AISI 4340 steel and AISI 4340M steel after quenching and low–temperature tempering [26,27], due to their similar alloy compositions and mechanical properties.

**Figure 8:**
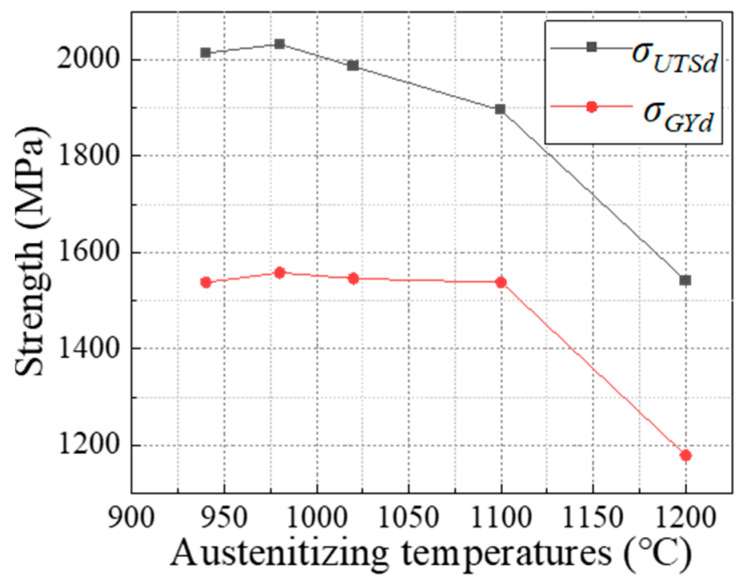
Dynamic yield strength and tensile strength at different austenitizing temperatures.

The calculated results are shown in Figure 8. From the figure, it can be observed that the dynamic ultimate tensile strength of the alloy steel is positively correlated with the maximum load. Between 940 °C and 1020 °C, the strength of the steel studied is approximately 2000 MPa, peaking at 2032 MPa at 980 °C. When the quenching temperature is increased to 1100 °C, the strength drops to 1895 MPa, and at a quenching temperature of 1200 °C, it rapidly decreases to 1542 MPa, a reduction of about 500 MPa compared to the peak at 980 °C.

## 4. Conclusions

1. At an austenitizing temperature of 980 °C, 35Si2MnCr2Ni3MoV steel achieves the optimal combination of strength and toughness. At this temperature, the steel’s impact energy is 67.2 J, and the dynamic tensile strength determined by instrumented impact test is 2032 MPa.
2. The studied steels show a slight decrease and then an increase in grain size with increasing temperature, with a pronounced coarsening after 1100 °C. Martensitic lath blocks and packets exhibit the same trend.
3. The proportion of high–angle grain boundaries is determined by the density of lath packets. The highest proportion occurs at 980 °C, providing the strongest resistance to crack propagation. As the solubility of stable austenite–forming elements increases with temperature, the amount of retained austenite significantly increases after 980 °C, reaching a peak of 4.7% at 1100 °C. After 1200 °C, as the lath packets coarsen and their density decreases, the probability of trapping retained austenite reduces.
4. 35Si2MnCr2Ni3MoV steel demonstrates superior crack initiation resistance compared to crack propagation resistance. As the austenitizing temperature increases, the fracture mode of 35Si2MnCr2Ni3MoV steel transitions from ductile dimple fracture to quasi–cleavage fracture.

## Figures and Tables

**Table 1:** Chemical composition of the studied steel (in wt.%).

Fe	C	Si	Mn	Cr	Ni	Mo	V
Balance	0.35	1.62	0.86	1.62	3.02	0.45	0.22

## Data Availability

Data are contained within the article.
